# Self-powered freestanding multifunctional microneedle-based extended gate device for personalized health monitoring

**DOI:** 10.1016/j.snb.2023.134788

**Published:** 2024-01-01

**Authors:** Rawan Omar, Miaomiao Yuan, Jing Wang, Majd Sublaban, Walaa Saliba, Youbin Zheng, Hossam Haick

**Affiliations:** a Department of Chemical Engineering and Russell Berrie Nanotechnology Institute, Technion - Israel Institute of Technology, Haifa 320003, Israel; b The Eighth Affiliated Hospital, Sun Yat-sen University, Shenzhen 518033, PR China; c Department of Electrical Engineering and Electronics, University of Liverpool, Liverpool L69 3GJ,United Kingdom

**Keywords:** Multifunctional sensor, Microneedles, Wearable sensor, Health monitoring, Self-powered

## Abstract

Online monitoring of prognostic biomarkers is critically important when diagnosing disorders and assessing individuals' health, especially for chronic and infectious diseases. Despite this, current diagnosis techniques are time-consuming, labor-intensive, and performed offline. In this context, developing wearable devices for continuous measurements of multiple biomarkers from body fluids has considerable advantages including availability, rapidity, convenience, and minimal invasiveness over the conventional painful and time-consuming tools. However, there is still a significant challenge in powering these devices over an extended period, especially for applications that require continuous and long-term health monitoring. Herein, a new freestanding, wearable, multifunctional microneedle-based extended gate field effect transistor biosensor is fabricated for online detection of multiple biomarkers from the interstitial fluid including sodium, calcium, potassium, and pH along with excellent electrical response, reversibility, and precision. In addition, a hybrid powering system of triboelectric nanogenerator and solar cell was developed for creating a freestanding, closed-loop platform for continuous charging of the device’s battery and integrated with an Internet of Things technology to broadcast the measurements online, suggesting a stand-alone, stable multifunctional tool which paves the way for advanced practical personalized health monitoring and diagnosis.

## Highlights

• A wearable self-powered multifunctional microneedle-based extended gate field effect transistor biosensor is fabricated.
• The sensor measures multiple biomarkers including sodium, calcium, potassium, and pH.
• The hybrid triboelectric nanogenerator and solar cell can continuously charge the system.
• This standalone wearable sensor holds promise as a personalized health monitoring tool.

## Graphical Abstract

**Figure:**
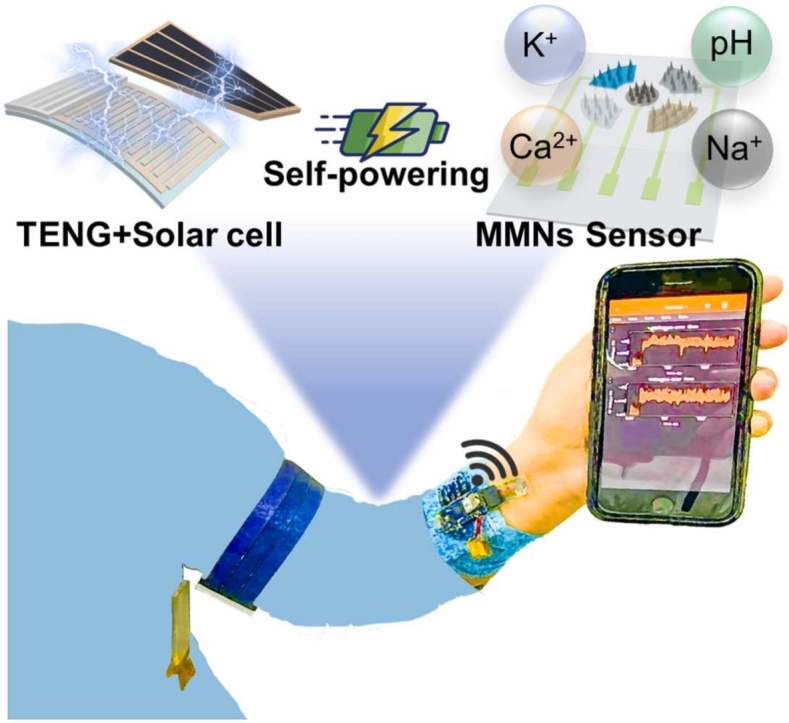

## 1 Introduction

Unforeseen outbreaks of pandemics and intensifications of chronic diseases drive the strong demand for advanced new technologies in the healthcare system, including advanced sensing technologies and self-powered wearable sensors. Wearable sensors have drawn much attention due to their user-friendliness and feasibility in monitoring and diagnosing health [1], [2], [3], [4]. Not only can they measure volatile organic compounds (VOCs), minerals, carbohydrates, and hormones from breath, skin, and body fluids, but they can also determine important biomarkers in interstitial fluid (ISF) like electrolytes such as potassium (K^+^), calcium (Ca^2+^), sodium (Na^+^) and more [5], [6], [7], [8], [9], [10], [11]. In this regard, using microneedles (MNs) based sensors is an effective strategy to monitor the ISF analytes in a minimally invasive and rapid manner, without the need for expert personnel or using painful and complex devices [6], [9], [12], [13], [14]. Measuring biomarkers including electrolytes and pH, is crucial due to their role in metabolic processes including regulating nerves, kidneys, heart, and muscle cells [15], [16], [17], [18], [19], [20], [21], [22], [23], [24]. pH was proven as a significant biomarker for monitoring health, given its impact on various biological processes. For instance, it is an effective biomarker in ISF for managing diabetes mellitus. Notably, pH serves as a biomarker to oversee and monitor wound healing progress [24], [25]. In addition, the deficiency or imbalance of electrolytes may lead to and/or indicate the prevalence of a wide spectrum of disorders such as cardiovascular diseases including myocardial infarction and arrhythmias, neural diseases, muscular dysfunction, kidney disorders, osteoporosis, and more. A single electrolyte measurement is not sufficient and informative for health analysis and diagnosis; thus, they are usually measured together for a more informative health assessment [14], [15], several studies reported the importance of measuring both K^+^ and Na^+^ electrolytes simultaneously since the correlation and ratio between them have a superior diagnostic advantage for the evaluation of several health cases, including acute myocardial infarction and acute kidney injury [16], [26], [27], [28].

At present, most biomarker-measuring gadgets in the market are either not wearable or lack an online feature. For example, LAQUAtwin devices can assess only a single type of biomarker, needing sweat, blood, or ISF extractions, while the HI97752 from Hanna Instruments company demands a large volume of liquid and is incompatible with blood or ISF samples with extra disturbing constituents [29], [30]. Additionally, in recent research, most of the online health monitoring sensors rely on sweat measurement, which is prone to contamination, evaporation and requires extensive sports, movements, or applying extraction methods to extract sweat. Others rely on measuring a sole type of parameter that cannot provide accurate and precise health evaluation [31], [32], [33], [34], [35], [36]. Another disadvantage is that there is still a significant challenge in powering wearable electronics over an extended period, especially for applications that require continuous monitoring. Therefore, for the devices to run for a reasonable time frame, battery and charging have to improve to transform the system into a closed-loop stand-alone device that can power itself enabling evolution to the next generation of wearables [37], [38], [39], [40], [41]. To deal with such issues, a wireless, battery-free, wearable sweat sensor was created, fueled by a triboelectric nanogenerator (TENG) [42]. Although this wearable platform can extract power from body motion through a flexible printed circuit board-based freestanding TENG, the single-form energy harvesting approach limits its energy output and it needs to accumulate energy to achieve a single measurement, and then needs to accumulate energy for the next measurement, which cannot truly realize real-time online monitoring. In other approaches, researchers utilized solar cell capabilities for self-powered sensors [43], [44]. Despite their advantages, including being a renewable energy resource and requiring little maintenance, solar cells are light-dependent, limiting their efficiency at night and in darkness. In addition, using self-powered MNs for multiple biomarker monitoring is not well established. Thus, advancing the wearable devices for multiple measurements particularly the ratio and combination of the biomarkers, besides being a stand-alone powered system can provide more precise information on the online health status. Additionally, advancing the sensors to rely on extended gate field effect transistors can provide an added advantage including stability and electrical performance advancing the obtained data [12], [13].

Herein, we developed a self-powered multifunctional MNs-based-extended gate field effect transistor (MMNs-EGFET) device to measure multiple biomarkers from the ISF, coupled with a hybrid self-powering system of TENG and a solar cell for a constant energy harvest and powering of a rechargeable battery. The hybrid energy harvesting system can fully utilize both solar energy and mechanical energy present around the human body converting them into electricity for powering wearable sensors. The platform is integrated with Internet of Things (IoT) technology to read and transfer the data to a smartphone in an online continuous manner , providing a freestanding all-in-one health diagnostic tool that can be applied in clinical, medical, and personal settings.

## 2 Experimental section

### 2.1 Fabrication of the MMNs

Poly(dimethylsiloxane) (PDMS) molds were prepared by engraving even holes using a laser cutter. The PDMS molds were added in tubes filled with 200 mg/mL polystyrene (PS) in dimethylformamide, then centrifuged at 3000 rpm for 5 min. The filled molds were dried at 80 °C. After demolding, the MMNs were spray-coated with silver nanowires (Ag NWs) after applying a mask. Each set of MNs was modified to fabricate each sensing layer with a specific selective composition for detecting Na^+^, K^+^, Ca^2+,^ and pH.

### 2.2 Fabrication of the TENG

The TENG was assembled using two parts. The first part is the substrate made of styrene-isoprene block copolymer (SIS) and transparent-coated polyethylene terephthalate (PET). A mask was applied on the PET and SIS film followed by spray coating Ag NWs. The mask was then removed to obtain the slide TENG. The other part was fabricated by carving a polytetrafluoroethylene (PTFE) film using the laser cutter.

This section is presented in detail in the Supporting Information.

## 3 Results and discussion

**Fig. 1:**
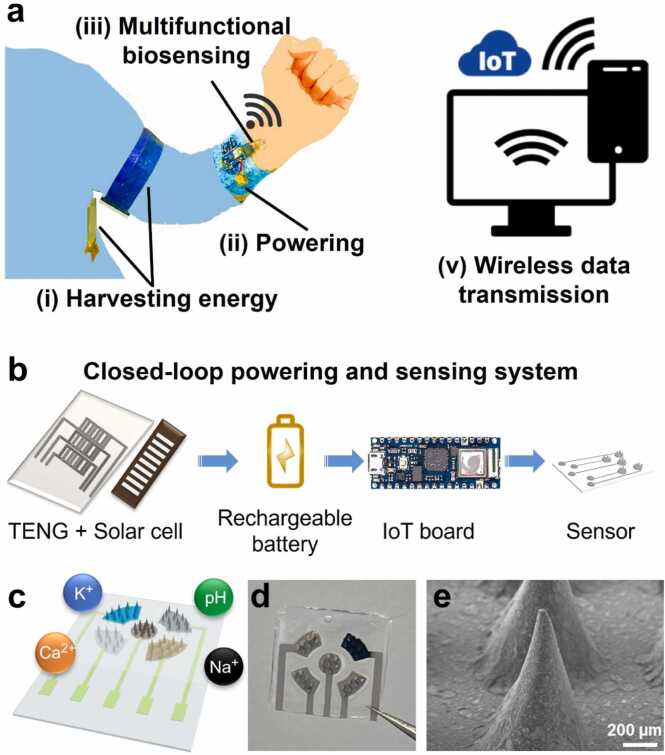
Overview of the wearable self-powered MMNs device for multiple biomarkers sensing and analysis. a Schematic of the wearable freestanding MMNs self-powered system. b Schematic of the components of the closed-loop powering and sensing system. c Schematic of the MMNs and the different biomarkers it can detect. d Photo of the MMNs. e Scanning electron microscope image of the MNs.

The developed wearable self-powered MMNs-EGFET device consists of different components that work harmoniously in a closed-loop mechanism. The energy is harvested naturally by body motion and light using a TENG coupled with a solar cell, and this energy constantly charges a researchable battery that powers a tiny Arduino board that we programmed to receive data outputs detected by a sensor that performs online biosensing of multiple biomarkers. The measured data is then transmitted constantly from the board using IoT technology to a smartphone\computer (Figs. 1a and 1b). The fabricated sensor is an MMNs-EGFET that is capable of detecting Na^+^, Ca^2+^, K^+^, and pH continuously and simultaneously from the ISF (Fig. 1c-e).

### 3.1 Concept, design, and fabrication of the MMNs-EGFET

The MMNs were designed and fabricated using molds of PDMS prepared using laser carving, followed by filling the template with a solution of PS, molding, and drying to obtain the rigid templated MMNs (Fig. S1, supporting information). The MMNs rigid PS substrate comprises 4 sensing areas and a shared reference. Ag NWs were spray coated on the MNs after applying a mask to gain conducive needles where each is conical with a tip radius equal to 10 µm, a base radium of 750 µm, and a height of 1000 µm. To enable the multiple biosensing, each sensing MNs area was modified with different selective modification depositions, namely, ion-selective membranes for Na^+^, Ca^2+^, K^+^, and polyaniline polymer (PANI) for pH detection. The reference (The middle MNs area) was fabricated by applying FeCl_3_ followed by a mixture of Polyvinyl butyral (PVB) and NaCl membrane [13], [24], [45]. Then, the MMNs array was connected as an extended gate to the FET for establishing the electrical connection (Fig. S2, supporting information). In this configuration, the drain-source voltage is constant and equal to 0.1 V, and the sensing electrodes are connected as extended gates to the FETs, while the gate voltage is scanned between 0 and 2 V through the reference electrode. Connecting the MMNs as an extended gate can offer added advantages including durability, fast response, and low-cost scalability [13], [46], [47].

### 3.2 Electrical and sensing performance of the MMNs-EGFET

**Fig. 2:**
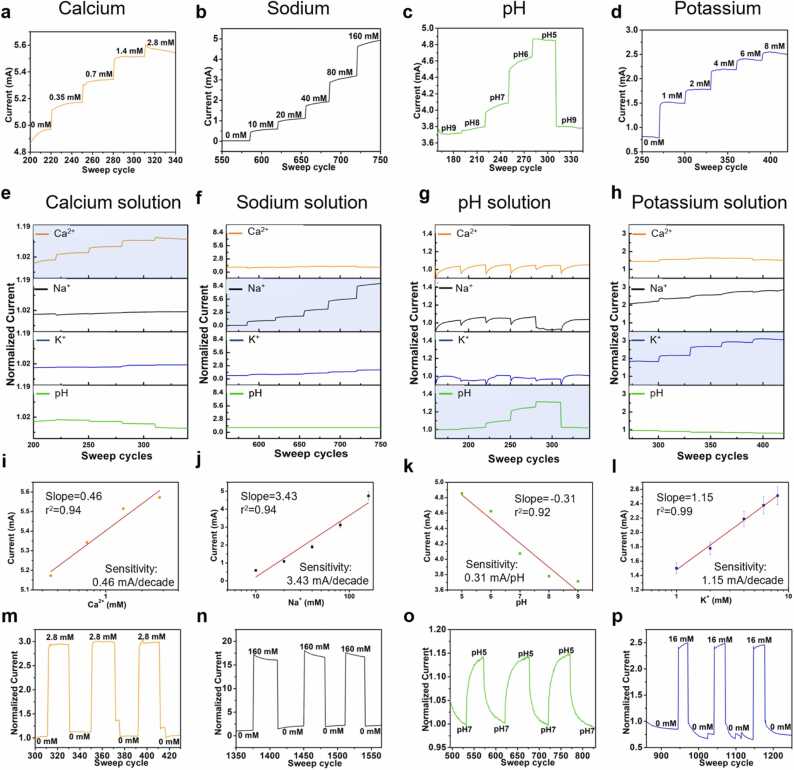
The electrical performance of the MMNs. Curves of the drain–source current of the sensors in response to elevated concentrations of a Calcium, b Sodium, c pH, d Potassium. Multiplex sensing and selectivity tests of all sensors using the solutions of e Calcium solution, f Sodium solution, g pH solution, h Potassium solution. Correlation curves of the sensors: i Calcium, j Sodium, k pH, l Potassium. Repeatability and reversibility of the MMNs: m Calcium, n Sodium, o pH, p Potassium.

The electrical and sensing performance of the MMNs-EGFET was tested by employing various solutions containing Na^+^, Ca^2+^, K^+^ and pH within the physiological range [21], [22], [48], [49]. Drain-source current elevated in reaction to the amplified concentrations of analytes for each individual sensor (one cycle equals 9 s, Fig. 2a-d). The concentration of calcium was 0–28 μM, sodium 0–160 mM, pH 9–5, and potassium 0–8 mM. In addition, the transfer curves of each one of the sensors including drain-source device current versus gate voltage are presented in Fig. S3 in the Supporting Information. Threshold voltages (Vth) were calculated based on these curves. The slopes were measured: the Ca^2+^ sensor displayed a Nernstian response of 20.11 mV/decade, exhibiting nearly Nernstian behavior, given the ideal value for divalent heavy metal ions is 29.58 mV/decade at 25 °C [50]. Similarly, both K^+^ (43.42 mV/decade) and pH (51.77 mV/decade) sensors demonstrated nearly Nernstian behavior, considering that the theoretical Nernstian response is 59.2 mV/decade at 25 °C [51], [52], [53]. The Na^+^ sensor exhibited a super Nernstian response, with a value of 104.33 mV/decade, and this is corroborated by its remarkably low limit of detection (LOD) of 0.56 µM (Fig. S4, Supporting Information). To prove the selectivity of the sensors and the joined answer of the MMNs-EGFET, the drain-source current was also measured in separate sets of solutions. It was noticed that each sensor responded exclusively to the goal analyte with assigned response steps, while the other sensors produced no answer whatsoever or reacted in unchanging steps, which implied that the sensors that reacted to the additional solutions did not offer a consistent stable answer to the distinct concentrations, presenting no precise pattern to trust (Fig. 2e-h). A linear correlation was formulated to evaluate the sensitivity of each sensor, and the sensitivities were computed by the incline and equated to 0.46 mA/decade, 3.43 mA/decade, 0.31 mA/pH, and 1.15 mA/decade for the Ca^2+^, Na^+^, pH, and K^+^ MNs biosensors respectively. A brief comparison of the MMNs-EGFET sensor and other reported sensors in the literature is presented in Table S1 in the Supporting Information. Additionally, the MMNs' repeatability and reversibility were verified for Ca^2+^, Na^+^, pH, and K^+^ sensors, showing first-rate reversibility when the concentration of each analyte was transformed from one concentration to another over three cycles (Fig. 2m-p). The sensors showed excellent baseline stability over time in hours, as demonstrated in Fig. S5 in the Supporting Information. Moreover, the long-term stability of the sensors was tested after three days and after one week, exhibiting robust electrical response and excellent long-term stability (Fig. S6 in the Supporting Information).

### 3.3 Electrical performance in artificial ISF solutions, skin validation, and biocompatibility tests

**Fig. 3:**
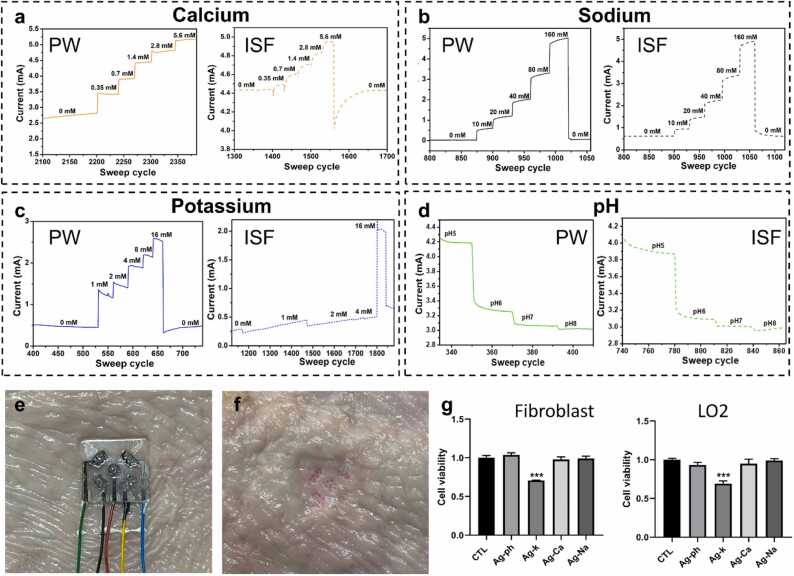
Electrical performance of the sensors in pure and artificial ISF solutions. a Calcium, b Sodium, c Potassium, d pH. e Photo of the MMNs sensor inserted on chicken skin. f Photo of the chicken skin after peeling off the MMNs sensor resulting in microholes. g The relative cell viability of Fibroblast and LO2 cells was tested by CCK-8 assay using different MNs. Error bars show standard deviations. Unpaired t-test. n = 3. Data are demonstrated as mean ± standard deviation (SD). ***p < 0.001.

To evaluate the electrical performance of the biosensors there’s a need for testing the performance in solutions similar to real-life settings. Thus, the sensors were tested in artificial ISF solutions, which included the basic ISF solution and an increased concentration of the target analytes. Each sensor was tested in both the pure solution including only the target biomarker and the artificial ISF solution to demonstrate a comparison of both. Fig. 3a-d demonstrates the drain-source curves of each MN sensor showing a similar trend and values in both cases along with excellent electrical and biosensing performance. Ca^2+^, Na^+^, and pH showed almost identical electrical performance in both types of solutions. K^+^ showed a similar current value but with slightly different steps stability which may be caused by the interference of other components in the solutions. The similar trend and current values show the excellent electrical performance of the sensors despite the co-founding components such as phosphates, minerals, and other ions that may influence the response. In all cases, the specific sensor could detect the target analytes and respond to the elevated concentrations with minimal drifting and influence on the sensing performance. To perform additional validation, the Na^+^ MN sensor was used to test with chicken skin and compared to a reference commercial device. To do so, chicken skin was cut into small slices of 2.5 cm × 3.0 cm, and each slice was conditioned with a different solution containing sodium in ISF with the concentrations 0 mM, 20 mM, 40 mM, and 160 mM for 24 h. Then, the surface of each slice was dried using kimwipes and the sensor was inserted into the skin causing micro-holes (Fig. 3e-f, Fig. S7, Supporting Information). The response of the sensor was measured after insertion on each one of the slices. The same solutions were also tested using a commercial device (Na^+^ meter, HORIBA, B-722) and as observed, both sensors provided a similar elevated trend of response to the change of concentrations. The sensor exhibited the expected logarithmic response shift when exposed to various skin samples with different concentrations, and the commercial device showed a linear increase in response with the elevated concentrations (Fig. S7, Supporting Information). Notably, the sensor demonstrated stability and sensing ability after four cycles of insertion and peeling-off from the different skin samples.

Biocompatibility and toxicity tests were performed to validate that the MMNs are biocompatible and don’t cause harm when used in on-body applications. The biocompatibility of the MMNs array was evaluated using fibroblasts and human fetal hepatocyte cell line (LO2) and was preliminarily evaluated by Cell Counting Kit-8 (CCK-8) assay. The MMNs array was divided into four parts, while each part represents the different sensors Ag-pH, Ag-Na^+^, Ag-K^+^, and Ag-Ca^2+^ to evaluate the effect of each sensing material separately. The cells were cultured with and without the sensor’s parts for 24 h and then the relative viability of the cells was studied by CCK-8 assay. The viability of both kinds of cells was higher than 95 % after being cultured in the solution with different MNs except K^+^ (Fig. 3g). Then, apoptosis assay and Live/Dead staining assay were carried out for the cultured cells with and without the sensors separately (n = 3 biological replicates). Furthermore, the Live/Dead staining of the cells (Fig. S8, Supporting Information) demonstrated that there are living cells (in green) with a very small number of dying cells (in red) after 24 h of cell culturing, also, in this case, in the K^+^ sensor the percentage of dead cells was higher. Apoptosis is a process of programmed cell death occurring in multicellular as a response to cell stimulation, like chemical products and pressure. Apoptosis results showed a similar apoptosis percentage of all cells incubated with the MN sensor’s parts compared to the control, whereas for K^+^ it was slightly higher at around 1.5 % (Fig. S8, Supporting Information). Overall, all results indicate that the sensors’ parts have excellent biocompatibility in-vitro except for the K^+^ sensing area. The lower cell viability and higher apoptosis of the K^+^ sensor can be explained by the fact that the sensing materials include the valinomycin antibiotic component, which has low biocompatibility compared to the other materials.[54] Thus, for future work, more optimizations have to be considered to elevate the biocompatibility of the K^+^ sensor.

### 3.4 Design and fabrication of the self-powered system

**Fig. 4:**
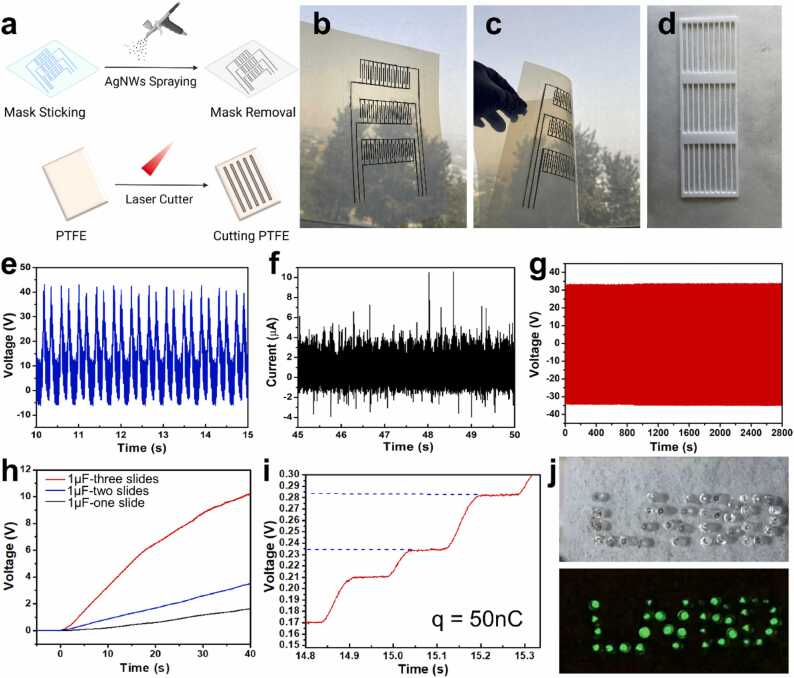
Fabrication and evaluation of the sliding-mode TENG. a Schematic illustration of the fabrication of the TENG. b-d Photos of the flexible slide TENG, including the flexible substrate with three slides Ag NWs and the counter slide of PTFE. e The output voltage of the three slides TENG. f The output current of the three slides TENG. g Long-term stability of the three-slide TENG over a period of time. h Charging curve using one slide, two slides, and three slides TENG. i Charge calculation of the three slides TENG. j Photos of lighting up the LEDs before and during the testing.

To create an all-in-one, convenient, and self-powered wearable device, a sliding-mode flexible TENG was designed and fabricated. By coupling triboelectrification and electrostatic induction, the sliding-mode TENG can harvest mechanical energy from in-plane movement (such as reciprocation) and convert it into electric power. Due to its reliance on harvesting energy from spontaneous body motion, the TENG provides a low-cost, easy-to-fabricate, and easy-to-use tool. In our case, natural body movement between the arm and waist of the human is an ideal energy source for powering the MMNs-EGFET device (Fig. S9, supporting information). TENG’s base substrate is composed of SIS and transparent PET coated with aluminum oxide/polyvinyl alcohol (PVA), providing flexibility and stretchability to the system. After that, a pattern of Ag NWs was sprayed with a mask onto the substrate, creating three sets of bendable and flexible slides (Fig. 4a-c, Video S1, Supporting Information). On the other hand, the counter slide was made from PTFE. To form the structure, a laser cutter was used to produce lines that correspond with the slides of Ag NWs on the substrate (Fig. 4d). Subsequently, it was validated that the electrical performance of the sliding-mode TENG could produce up to 40 V on average (Fig. 4e), and a current that averaged 4 μA (Fig. 4f). To demonstrate its long-term stability, the system was kept operating for an hour, exhibiting excellent repeatability and stability (Fig. 4g). Additionally, to emphasize the importance of the three sets of slides, output voltages were collected for one slide (black curve), two slides (blue curve), and three slides (red curve), clearly illustrating that three slides yielded higher output than one and two slides (Fig. 4h). From this, the charge produced from the three slides TENG was calculated to be 50 nC/cycle (Fig. 4i). To demonstrate the device’s effective charging capacity, it was used to light 36 light-emitting diodes (LEDs) (Fig. 4j). To showcase its application for wearable devices, the TENG was used to power the LEDs employing a mechanical motor mimicking walking, and by a human's motion as well (Videos S2 and S3, Supporting Information).

Supplementary material related to this article can be found online at doi:10.1016/j.snb.2023.134788.

The following is the Supplementary material related to this article Video S1, Video S2 and Video S3.

Video S1

Video S2

Video S3.

### 3.5 Hybrid energy-harvesting for freestanding MMNs self-powered IoT device

**Fig. 5:**
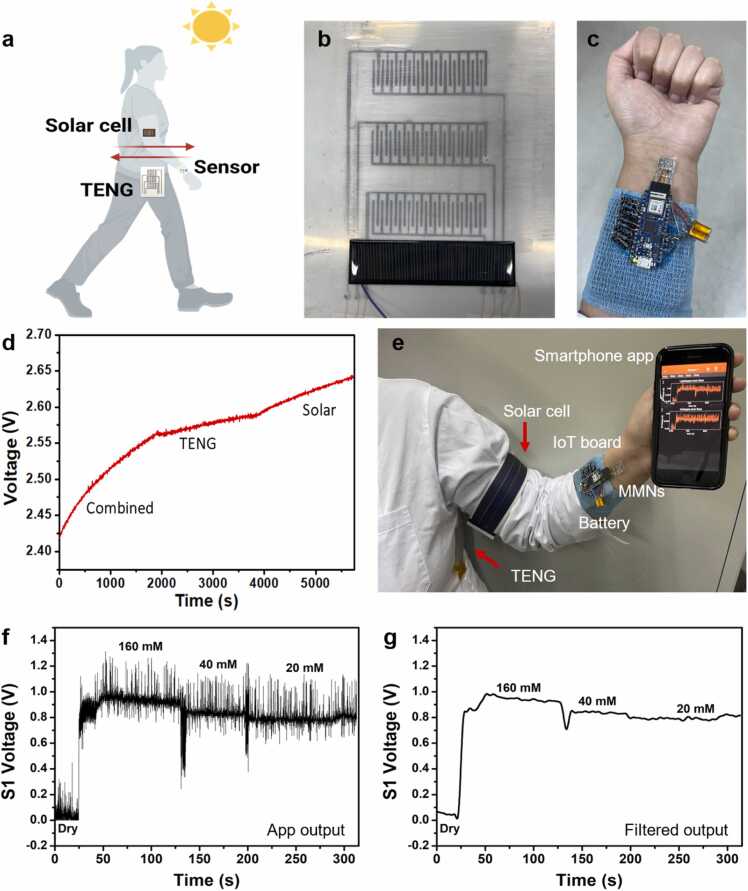
A freestanding MMNs-TENG powered IoT device. a Schematic of the hybrid energy-powering system. b Photo of the hybrid energy-powering system, including the TENG and solar cell. c Photo of the wearable MMNs-TENG device. d Charging capability of the hybrid combined powering system, charging with TENG only, and charging with solar cell only. e Photo of the wearable freestanding MMNs-TENG powered IoT device for measuring biomarkers and transmitting the data to the smartphone app. f, g Original and filtered output of Na^+^ readings from the smartphone app.

To enhance and accelerate the energy-harvesting capability for charging, a hybrid energy harvesting system made of slide TENG and a solar cell was developed. Despite sole TENGs and solar cells' effectiveness in energy harvesting, using a single-form energy harvesting approach limits its output because it requires time and external conditions to accumulate energy to achieve a single measurement. Using TENGs requires continuous body movement, whereas solar cells require prolonged sunlight, so using each separately will be limited in case of lack of movement or darkness. Thus, in addition to the TENG, a solar cell (dimensions 2.7 cm × 9.9 cm) was included, enabling the device to collect energy cost-effectively and conveniently from both body movement and solar power (Fig. 5a-b). The current and voltage output performance of the solar cells in dark and light conditions are presented in Fig. S10 in the Supporting Information. A TENG is affixed to the waist of the wearer, while a counter slide is mounted on the inner arm. As the wearer moves, friction between the two parts will generate an electrical output. The solar cell harnesses light and solar energy, transforming it into electricity, allowing both parts to charge autonomously or collectively for more efficient and cost-effective power production.

To construct a closed-loop system, a low-power consumption Arduino IoT board (a Nano 33 IoT based on the SAMD21 microcontroller, consuming around 10–20 mA of current in active mode) was programmed and connected to the MMNs-EGFET sensors and the hybrid energy-generating system, integrating both parts via the IoT device. An Arduino code was composed to read the data generated by the MMNs-EGFET and transfer and present it in a mobile application (Figs. S11, S12 and S13, Supporting Information). A wearable, self-standing device that can be affixed to the arm and record data using the MMNs-EGFT was assembled (Fig. 5c). The energy-generating system was connected and employed to recharge the rechargeable battery (nominal capacity: 30 mhA), which powered the IoT board and signaled that the board was functioning by blinking yellow.

To evaluate the performance of the combined system, consisting of both a TENG and a solar cell, and the performance of each of these components when used separately, their respective efficiencies in charging a rechargeable battery were compared. It was found that the battery was charged more quickly and effectively when the two components were combined, with an elevated charging slope when compared to each individual component (Fig. 5d).

This wearable self-powered device, featuring multi-modal nanosensors (MMNs-EGFET), a rechargeable battery, an IoT board, and a hybrid powering system, served as a closed-loop platform for the detection of biomarkers and transmission of the associated data to the smartphone app of the wearer (Fig. 5e). The device demonstrated the capability to measure multiple biomarkers while maintaining its continuous power through the rechargeable battery powered by the hybrid energy-powering system. Examples of these outputs for Na^+^ readings can be seen in Fig. 5f-g. The reading outputs of the rest of the biomarkers from the smartphone app, K^+^, Ca^2+^ and pH are presented in the Supporting Information (Fig. S14, Supporting Information). Notably, the MMNs responded to the specific target analyte with the expected trend, demonstrating elevated response upon elevating the concentration of the electrolytes, K^+^ and Ca^2+^, and decreasing response upon elevated pH values.

The effectiveness, convenience, and speed of this self-sustaining MMNs-EGFET device for health monitoring provide an exciting potential for it to serve as a low-cost, closed-loop, self-sustaining platform, and as a new fundamental technique for medical, personal, and tailored health surveillance.

## 4 Conclusions

This work demonstrates a wearable, freestanding MMNs-based self-powered IoT sensing device for online health monitoring. The device is composed of an MMNs-EGFET biosensor to detect multiple biomarkers including Na^+^, K^+^, Ca^2+^, and pH, which is coupled with an IoT device powered by a hybrid powering system based on TENG and solar cell. The MMNs-EGFET sensor demonstrated exceptional electrical performance towards the target analytes, displaying excellent selectivity, signal repeatability, long-term stability, and high sensitivity: 0.46 mA/decade for Ca^2+^, 3.43 mA/decade for Na^+^, 0.31 mA/pH for pH, and 1.15 mA/decade for K^+^. Moreover, the hybrid powering system exhibited outstanding electrical performance. The sliding-mode TENG yielded an average output voltage of up to 40 V and an average current of 4 μA, while the solar cell produced an output voltage of 4 V and an average output current of 330 μA.The IoT device receives the measurements of the MMNs sensor and transmits the data to a smartphone app. This platform suggests a freestanding wearable device for overall personalized health monitoring, paving the way to the next generation of stand-alone wearables for clinical and personal use.

## CRediT authorship contribution statement

**Rawan Omar:** Conceptualization, Methodology, Investigation, Visualization, Software, Formal analysis, Writing – original draft. **Miaomiao Yuan:** Investigation, Resources, Writing – review & editing. **Jing Wang:** Investigation, Writing – review & editing. **Majd Sublaban:** Investigation, Software. **Walaa Saliba:** Resources, Software. **Youbin Zheng:** Investigation, Conceptualization, Visualization, Writing – review & editing, Supervision. **Hossam Haick:** Conceptualization, Resources, Writing – review & editing, Funding acquisition, Supervision.

## Declaration of Competing Interest

The authors declare that they have no known competing financial interests or personal relationships that could have appeared to influence the work reported in this paper.

## Data Availability

Data will be made available on request.

## Footnotes

Appendix A Supplementary data associated with this article can be found in the online version at doi:10.1016/j.snb.2023.134788.
