# Supplementary material for: Self-powered freestanding multifunctional microneedle-based extended gate device for personalized health monitoring
Source: Sens Actuators B Chem. 2024 Jan 1;398:134788. doi: 10.1016/j.snb.2023.134788 (PMC10652171; doi:10.1016/j.snb.2023.134788)
Supplement: Table S1 — Supplementary material [file mmc1.docx]

Supporting Information

Self-powered freestanding multifunctional microneedle-based extended gate device for personalized health monitoring

Rawan Omar ^a^, Miaomiao Yuan ^b^, Jing Wang ^a^, Majd Sublaban ^a^, Walaa Saliba ^a^, Youbin Zheng ^a,c*^, Hossam Haick ^a*^

^a^  Department of Chemical Engineering and Russell Berrie Nanotechnology Institute, Technion - Israel Institute of Technology, Haifa 320003, Israel.

^b^ The Eighth Affiliated Hospital, Sun Yat-sen University, Shenzhen 518033, P. R. China.

^c^ Department of Electrical Engineering and Electronics, University of Liverpool, Liverpool L69 3GJ,

United Kingdom.

* E-mails: hhossam@technion.ac.il, youbin.zheng@liverpool.ac.uk

KEYWORDS: multifunctional sensor, microneedles, wearable sensor, health monitoring, self-powered

**Contents:**

**Materials and Methods**

**Supplementary Figures**

Supplementary Figure 1 | The fabrication process of the multifunctional microneedles (MMNs). (a) Schematic of the fabrication procedure of the MMNs. (b) SEM image of the deposited silver nanowires (Ag NWs) on the MN. (c) SEM image of the MN tip.

Supplementary Figure 2 | The electrical connection of the MMNs-based extended gate field transistor (MMNs-EGFET).

Supplementary Figure 3 | Transfer curves of the sensors, drain-source device current versus gate voltage.

Supplementary Figure 4 | Threshold voltage response curves.

Supplementary Figure 5 | Sensors’ stability over time in hours.

Supplementary Figure 6 | The long-term stability of the sensors, including the original response of the sensors (Day 1), the response after 3 days (Day 3) and the response after one week (Day 7).

Supplementary Figure 7 | Validations using chicken skin. (a, b) Chicken skin cuts, conditioned with several concentrations. (c) Photo of the skin after inserting the MMNs. (d) The response of the MMNs sensor to sodium in comparison with the commercial device.

Supplementary Figure 8 | (a) In vitro biocompatibility evaluation of the MMNs divided array by apoptosis assay. (b) Apoptotic cells of Fibroblast and LO2 cell lines that were determined by flow cytometry after 24 hours of culturing with/without the MMNs. (c) The Live/Dead staining assay was used to evaluate the toxicity of MNs. Live (Green), Dead (Red). The apoptosis of cells was checked after co-incubation with MNs for 24 h. Unpaired t-test. n=3. Data are presented as mean values ± SD. ***p< 0.001.

Supplementary Figure 9 | Slide- triboelectric nanogenerator (TENG) work mechanism.

Supplementary Figure 10 | The output performance of the solar cell in dark and light conditions. (a) The output voltage. (b) The output current.

Supplementary Figure 11 | Circuit diagram of the IoT board for combining the powering system and the MMNs.

Supplementary Figure 12 | Powering the IoT system using the recharged battery and connecting to the smartphone app.

Supplementary Figure 13 | Components of the IoT board for combining the MMNs and the powering system (TENG + Solar cell).

Supplementary Figure 14 | Original and filtered output of the biomarkers’ readings from the Smartphone app.

**Supplementary Table 1**

**Supplementary Videos 1-3**

**Materials and Methods**

**Materials**

Polyvinyl butyral (PVB), polyvinylpyrrolidone (PVP), polycaprolactone (PCL), styrene-isoprene block copolymer (SIS), poly (vinyl chloride) (PVC), polyvinyl alcohol (PVA), and Dulbecco's Phosphate-Buffered Saline (DPBS) were purchased from Sigma Aldrich (St. Lous, MO). Transparent coated polyethylene terephthalate (PET) substrate was purchased from Mitsubishi Paper Mills Limited (Tokyo, JAPAN). Penicillin-Streptomycin 10X was purchased from Biological Industries (Beit-Haemek, Israel). Resistors, capacitors, batteries, and the Arduino board were purchased from Lion Electronics Ltd., Talmir Electronics Ltd. (Haifa, Israel) and 4Project (Yehud, Israel). The Live/Dead staining assay was purchased from Thermo Fisher Scientific (Waltham, MA, USA). CCK-8 was obtained from Dojindo Molecular Technologies (Japan). Annexin V-FITC/PI apoptosis kit was brought from BD (USA). The Dulbecco’s Modified Eagle’s Medium (DMEM) and fetal bovine serum (FBS) were purchased from Gibco (USA). All chemical solutions were purchased from Bio-Lab Ltd (Jerusalem, Israel), without any further purification before use. Purified water was used for the preparation of reagents and synthesis. All solutions were prepared using Milli-Q water (18.2 MΩ cm, Millipore, Bedford, MA, USA).

**Chemical and Electrical Characterization**

Keithley 2536A System Source meter was used to measure and test the electrical performance of the MMNs-EGFET biosensors. Zeiss Ultra Plus High-Resolution Cryo-Scanning Electron Microscope (HR-Cryo-SEM) was used to characterize the synthesized materials and fabricated sensors. A laser cutter, Universal Laser Systems VersaLASER (VLS), was used to make the sensors' designs, masks, and shapes.

**Synthesis of Silver Nanowires (Ag NWs)**

2.5 gr PVP was added to 40 mL ethyl glycol, dissolved and mixed with 100 µL 0.15 M FeCl_3_ dissolved in ethyl glycol at 160°C. Then, 100 µL of NaCl 0.15M was added for the reaction. Afterward, 10 mL 1.5 M AgNO_3_ was dropwise during mixing until the color of the mixture changed from brown to light silver. To stop the reaction, methanol was added after 2 h. The samples were centrifuged to separate from the other residues and then dried at 60°C using a vacuum oven to obtain the final Ag NWs clean product. [1,2] Then, 50 mg of dried Ag NWs were suspended in 5 mL chloroform solution to get 10 mg/mL Ag nanowire solution. Following this, 150 μL of PCL solution (50 mg/mL in chloroform) was added into the 10 mg/mL Ag NWs solution and sonicated for 5 min before use.

**Fabrication of the Multifunctional Microneedles (MMNs)**

Poly(dimethylsiloxane) (PDMS) molds were prepared by carving holes using a laser cutter based on the desired shape. The created PDMS molds were added to tubes filled with 200 mg/mL polystyrene in dimethylformamide, and then centrifuged (3000 rpm for 5 min). The filled molds were dried overnight at 80 °C. After demolding, the multifunctional microneedles (MMNs) were spray-coated with Ag NWs after applying a mask.

**Fabrication of the Shared Reference**

10 μL 0.05M FeCl_3_ solution was drop cased on the middle MNs reference electrode for 1 min, then washed with purified water and dried. NaCl and PVB solution in methanol was drop-casted followed by drying for 30 min at room temperature to coat the reference electrode. [2–4]

**Fabrication of pH Sensor**

A powder of polyaniline (PANI) was mixed in isopropyl alcohol (10mg/mL), prepared with 3% PVA, and then spray coated homogeneously on one of the sensing MNs sensing areas. The sensor was then covered with a Nafion membrane to preserve the stability of the sensing materials.

**Fabrication of Na^+^ Sensor**

Na ionophore X (10 mg) was mixed with PVC (33 mg), sodium tetrakis[3,5-bis(trifluoromethyl)phenyl] borate (Na-TFPB) (5.5 mg), and bis(2- ethylehexyl) sebacate (DOS) (654.5 mg) and then dissolved and mixed in 6.6 mL tetrahydrofuran (THF). The solution was stored overnight at 4 °C and then was dropped on one of the MNs sensing areas. The sensor was then covered with a Nafion membrane to preserve the stability of the sensing materials.

**Fabrication of K^+^ Sensor**

Valinomycin (2%, w/w) was mixed with NaTPB (0.5%), DOS (64.7%, w/w), and PVC (32.7%, w/w), then dissolved and mixed in cyclohexanone. The solution was stored at 4 °C overnight and then was drop-casted on one of the MNs sensing areas. The sensor was then covered with a Nafion membrane to preserve the stability of the sensing materials.

**Fabrication of Ca^2+^ Sensor**

A mixture of 100 mg was prepared by 1 wt% calcium ionophore II, 33 wt% PVC, 0.5 wt% Na-TFPB, and 65.5 wt% DOS, then mixed and dissolved in 660 μl THF. The solution was stored at 4 °C overnight and then was drop-casted on one of the MNs sensing areas. The sensor was then covered with a Nafion membrane to preserve the stability of the sensing materials.

**Preparation of Artificial Interstitial Fluid (ISF) Solution**

A stock solution of artificial interstitial fluid (ISF) was created by adding 0.495 g glucose (C_6_H_12_O_6_), 1.265 g saccharose (C_12_H_22_O_11_), 0.09 g monosodium phosphate (Sodium phosphate monobasic NaH_2_PO_4_) (or 0.1035g NaH_2_PO_4_*H_2_O), 0.14 g calcium chloride (CaCl_2_), 1.19 g HEPES (C_8_H_18_N_2_O_4_S), 0.04 g magnesium sulfate (MgSO_4_), 3.59 g sodium chloride (NaCl), 0.13 g potassium chloride (KCl) to 0.5 L deionized water and mixed properly. The solution was adjusted to pH 7 and then stored at 4 °C. It’s important to note that the basic ISF solution prepared for each one of the electrolytes that was used for a baseline, didn’t include the target tested analyte for the specific sensor. e.g. the basic ISF solution for testing the potassium sensor didn’t include 0.13 g of KCl.

**Cell Culture**

Fibroblast cells and LO2 cells derived from human normal liver were obtained from the American Type Culture Collection (ATCC). Cells were cultured in DMEM culture media supplemented with 10% FBS, 1% penicillin-streptomycin, and 0.1% 2-mercaptoethanol at 37 °C and 5% CO_2_.

**Cell Viability**

CCK-8 was used to assess cell viability. The MMNs were divided into four parts that included each sensing area separately and then were washed with PBS three times. In brief, fibroblast cells or LO2 cells suspension was cultured in 48-well plates (2×10^4^ cells/well), followed by treating with the different MNs for 24 h, respectively. Then, 20 μL of CCK-8 was added for 1 h before using an ELx800 Absorbance Microplate Reader to read the optical density at 450 nm wavelength.

**Cell Apoptosis**

Fibroblast cells and LO2 cells were plated on a 6-well at a density of 50%. After 12 h, the divided MNs were added into the well and co-cultured for 24 h. Cells were collected and stained by apoptosis kit and then detected using flow cytometry (BD Biosciences). The data was analyzed using flow software.

**Live/Dead Staining**

Fibroblast cells and LO2 cells were plated and cultured (1 × 10^5^ cells/well) in 6-well overnight before being treated with the MNs for 24 h. Then, the cells were washed with PBS twice and stained using the Live/Dead Cell Imaging Kit for 30 min. Afterwards, the cells were washed with PBS three times, and the living cells and dead cells were photographed using fluorescence microscopy (Nikon ECLIPSE Ti-U, Japan).

**Fabrication of the Triboelectric Nanogenerator (TENG)**

The triboelectric nanogenerator (TENG) is composed of two parts. For fabricating the first part, a mask with a shape of three slides was prepared using a laser cutter and was applied on a PET and SIS film followed by spray coating Ag NWs. The mask was then removed to obtain the slide TENG. The other part was fabricated by carving a PTFE film using the laser cutter.

**Statistical Analysis**

Quantitative data are presented as mean ± SD. Statistical differences were estimated using One-way ANOVA statistical analysis when p < 0.05 values were considered statistically significant.

**Ethical Statement**

The medical research ethics committee of the Eighth Affiliated Hospital, Sun Yat-sen University (Futian, Shenzhen)-ZDFBKYLL 2021-024-02 supervised the human experiments conducted in this study. The volunteers who participated in the on-body demonstrations provided their informed consent.

**Supplementary Figures**

**
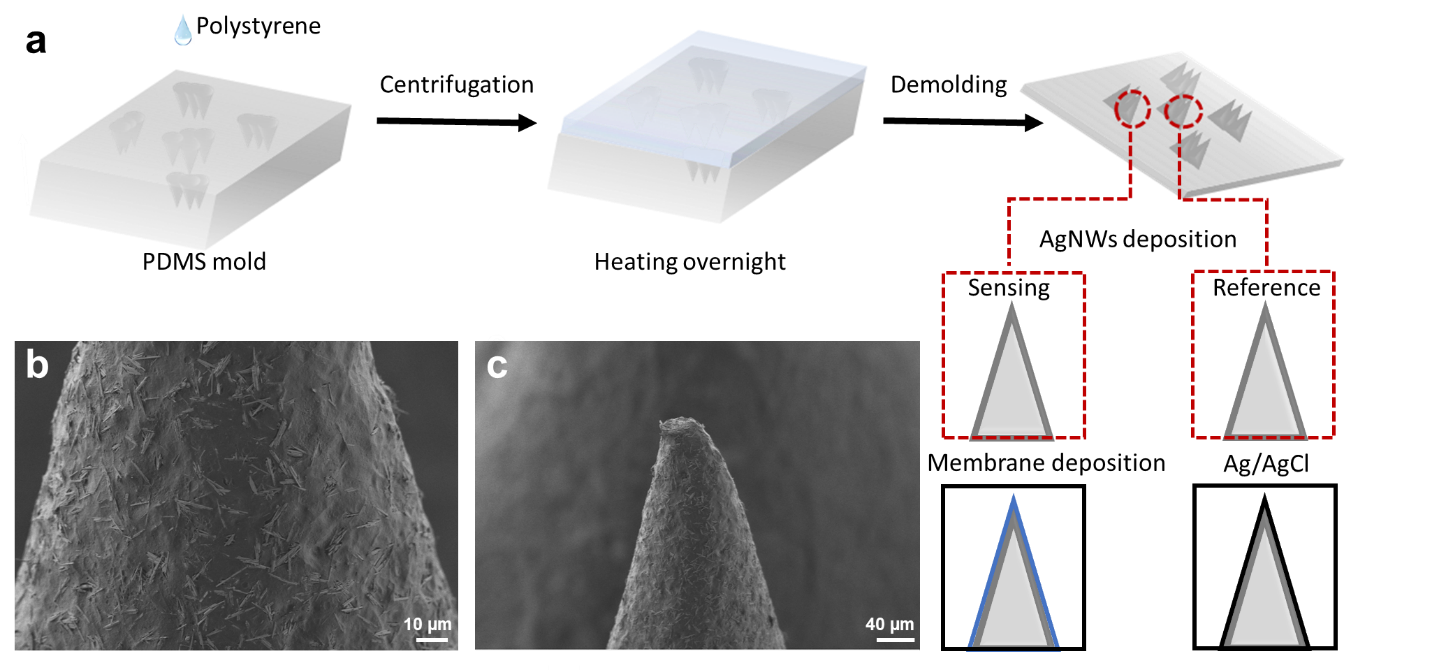
**

Supplementary Figure 1 | The fabrication process of the multifunctional microneedles (MMNs). (a) Schematic of the fabrication procedure of the MMNs. (b) SEM image of the deposited silver nanowires (Ag NWs) on the MN. (c) SEM image of the MN tip.


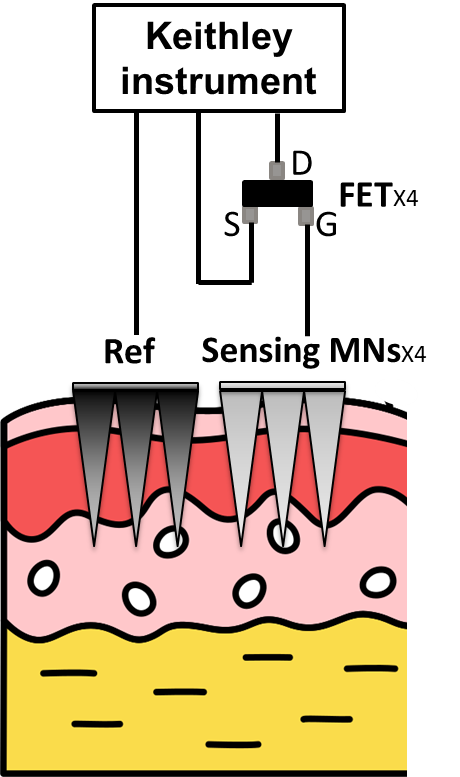


Supplementary Figure 2 | The electrical connection of the MMNs-based extended gate field transistor (MMNs-EGFET).


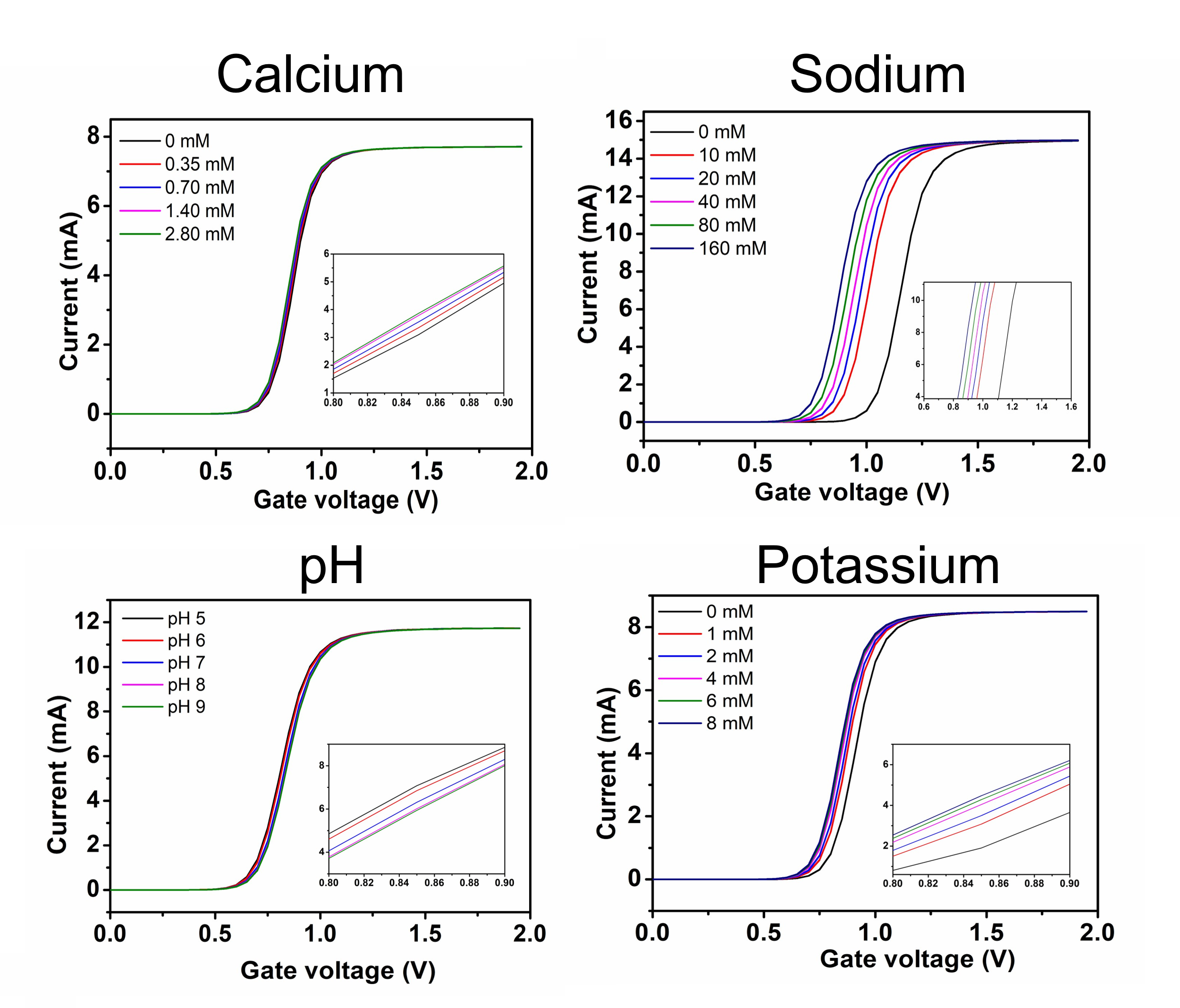


Supplementary Figure 3 | Transfer curves of the sensors, drain-source device current versus gate voltage.


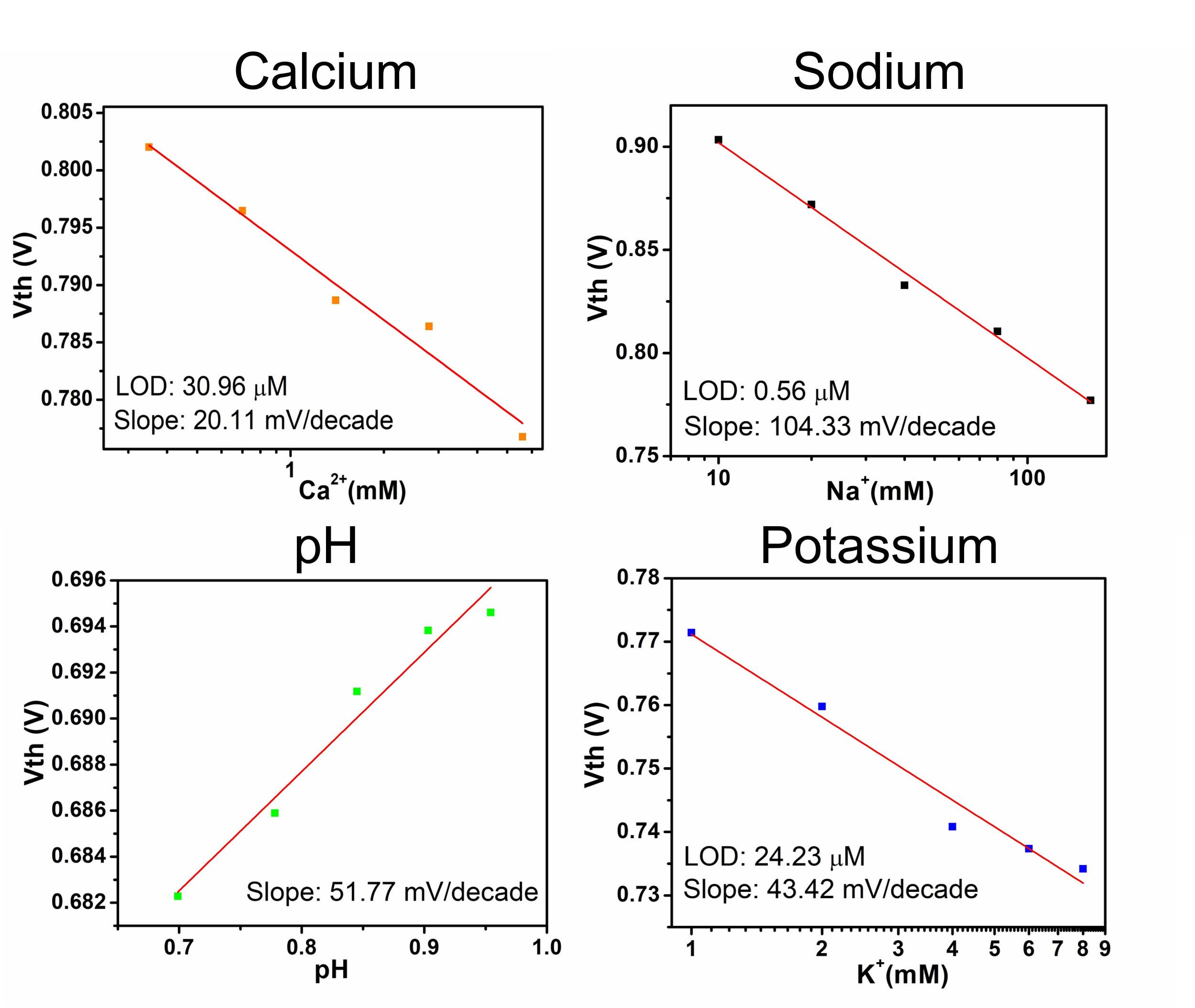


Supplementary Figure 4 | Threshold voltage response curves.


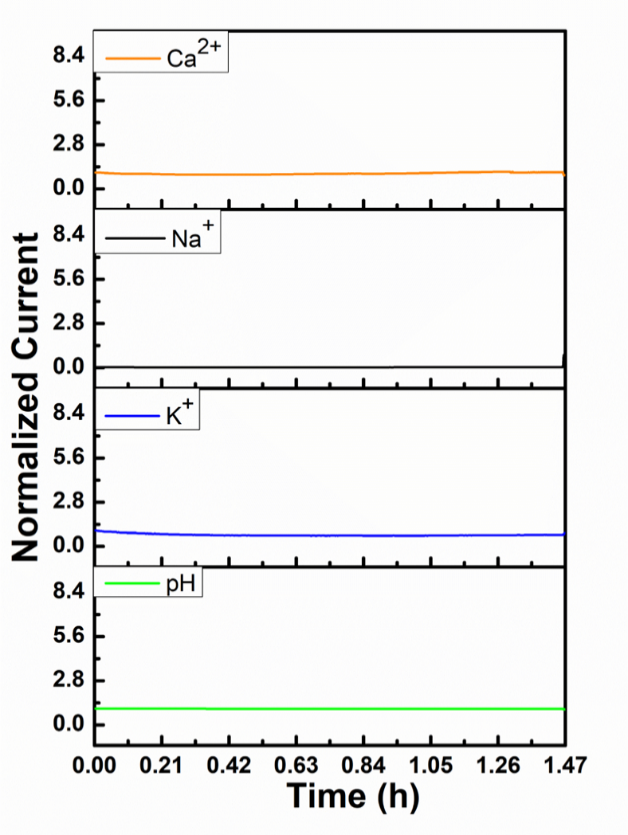


Supplementary Figure 5 | Sensors’ stability over time in hours.


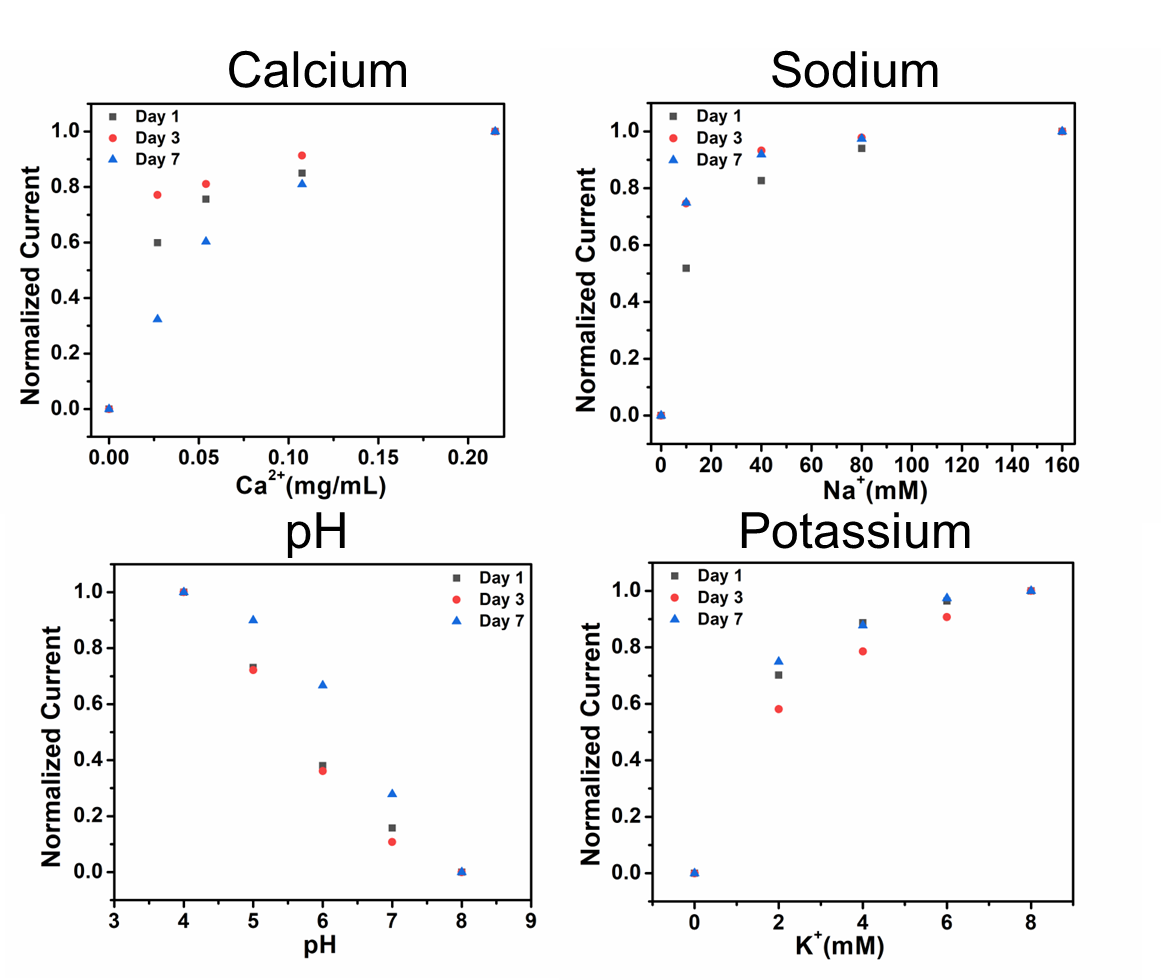


Supplementary Figure 6 | The long-term stability of the sensors, including the original response of the sensors (Day 1), the response after 3 days (Day 3) and the response after one week (Day 7).


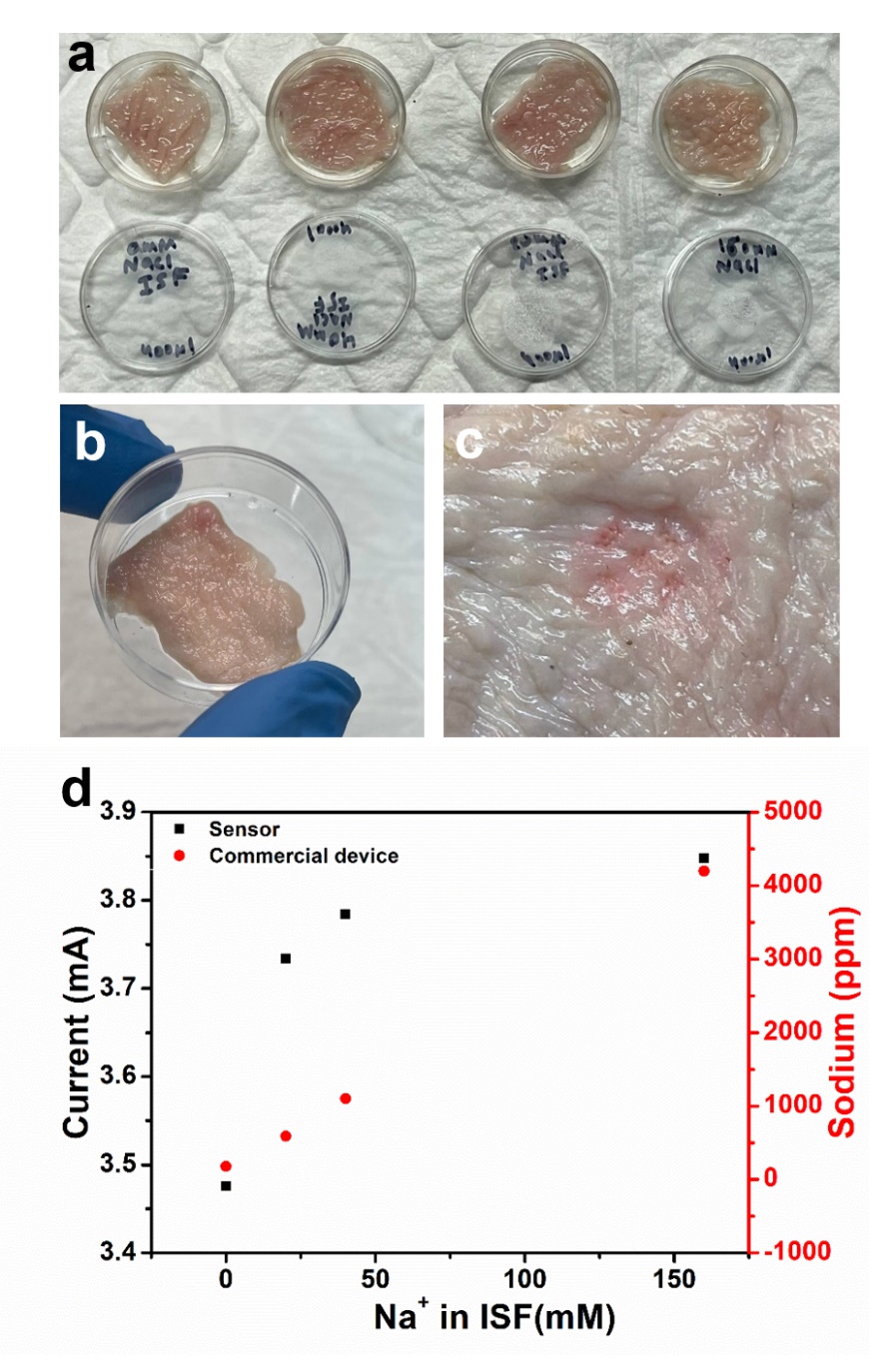


Supplementary Figure 7 | Validations using chicken skin. (a, b) Chicken skin cuts, conditioned with several concentrations. (c) Photo of the skin after inserting the MMNs. (d) The response of the MMNs sensor to sodium in comparison with the commercial device.


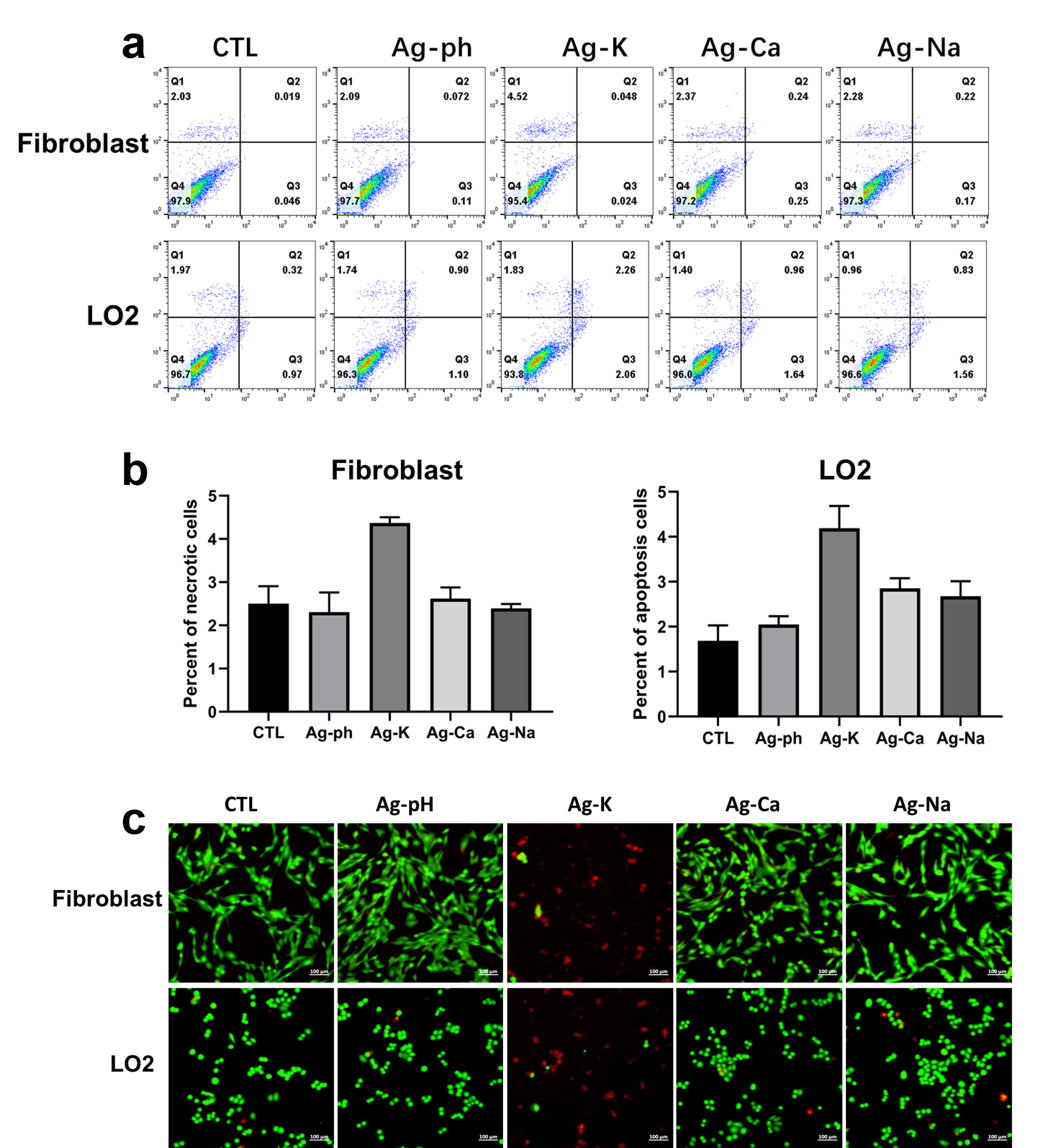


Supplementary Figure 8 | (a) In vitro biocompatibility evaluation of the MMNs divided array by apoptosis assay. (b) Apoptotic cells of Fibroblast and LO2 cell lines that were determined by flow cytometry after 24 hours of culturing with/without the MMNs. (c) The Live/Dead staining assay was used to evaluate the toxicity of MNs. Live (Green), Dead (Red). The apoptosis of cells was checked after co-incubation with MNs for 24 h. Unpaired t-test. n=3. Data are presented as mean values ± SD. ***p< 0.001.


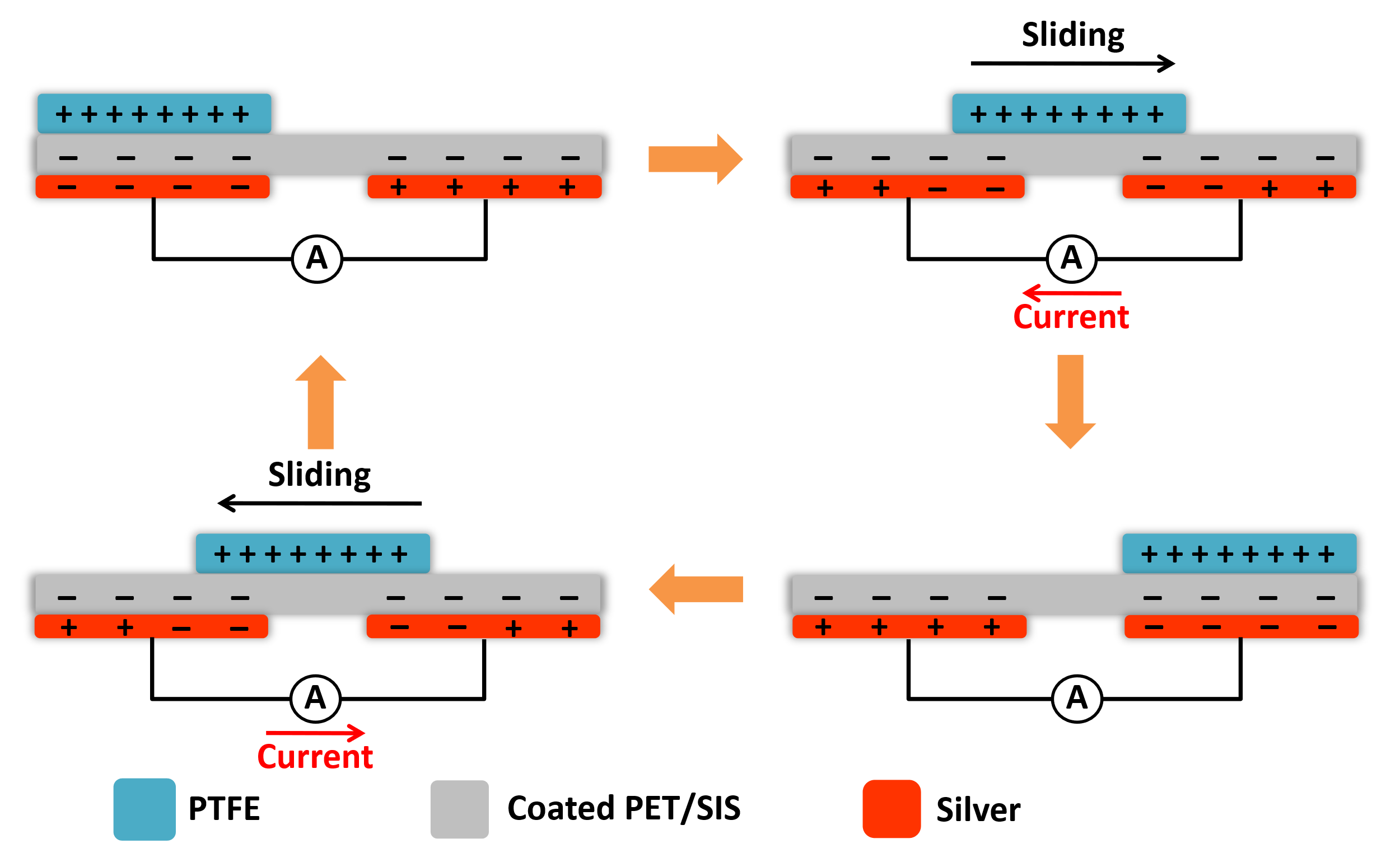


Supplementary Figure 9 | Slide- triboelectric nanogenerator (TENG) work mechanism.


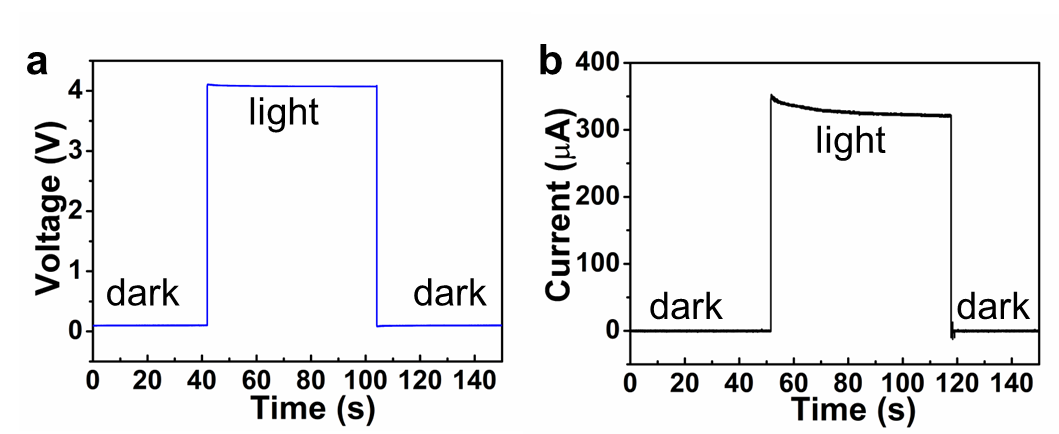


Supplementary Figure 10 | The output performance of the solar cell in dark and light conditions. (a) The output voltage. (b) The output current.


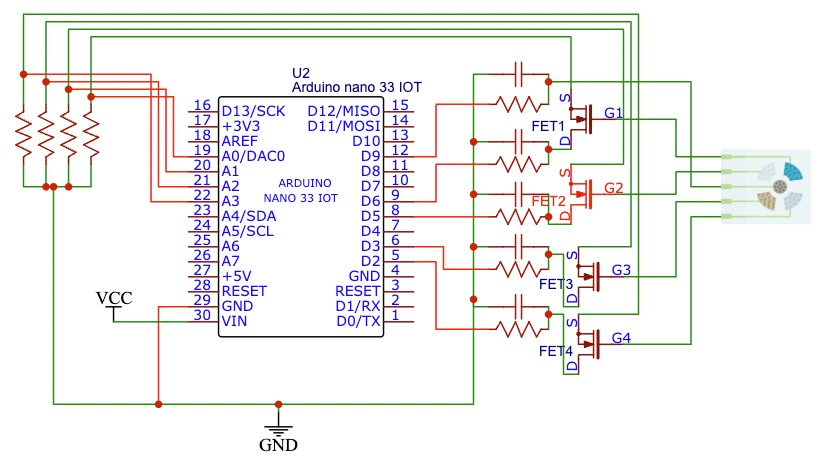


Supplementary Figure 11 | Circuit diagram of the IoT board for combining the powering system and the MMNs.


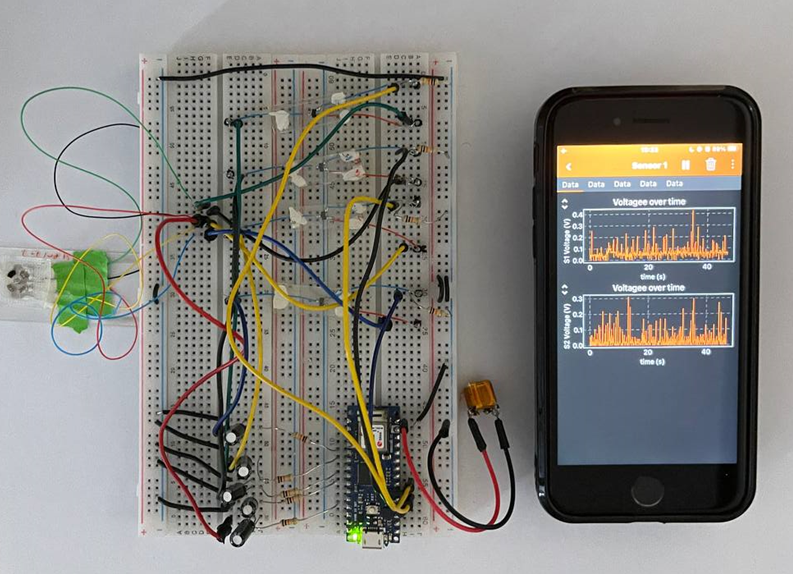


Supplementary Figure 12 | Powering the IoT system using the recharged battery and connecting to the smartphone app.


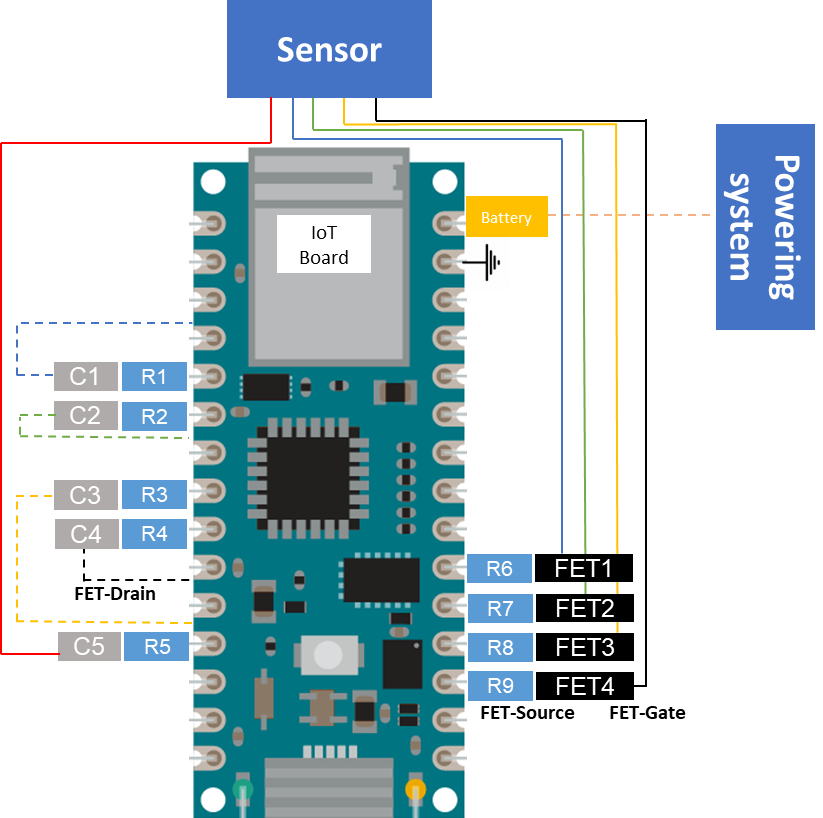


| **Component** | **Value** | **Description** |
| --- | --- | --- |
| IoT Board | SAMD21 Cortex®-M0+ 32bit low power ARM MCU | Arduino Nano 33 IoT |
| FET1-FET4 | N-Channel 1.25-W, 2.5-V MOSFET | Field Effect Transistors |
| R1-R9 | 10 KΩ | Resistors |
| C1-C5 | 22 µF | Capacitors |
| Battery | 7.4 V, 30 mhA | Rechargeable battery |

Supplementary Figure 13 | Components of the IoT board for combining the MMNs and the powering system (TENG + Solar cell).


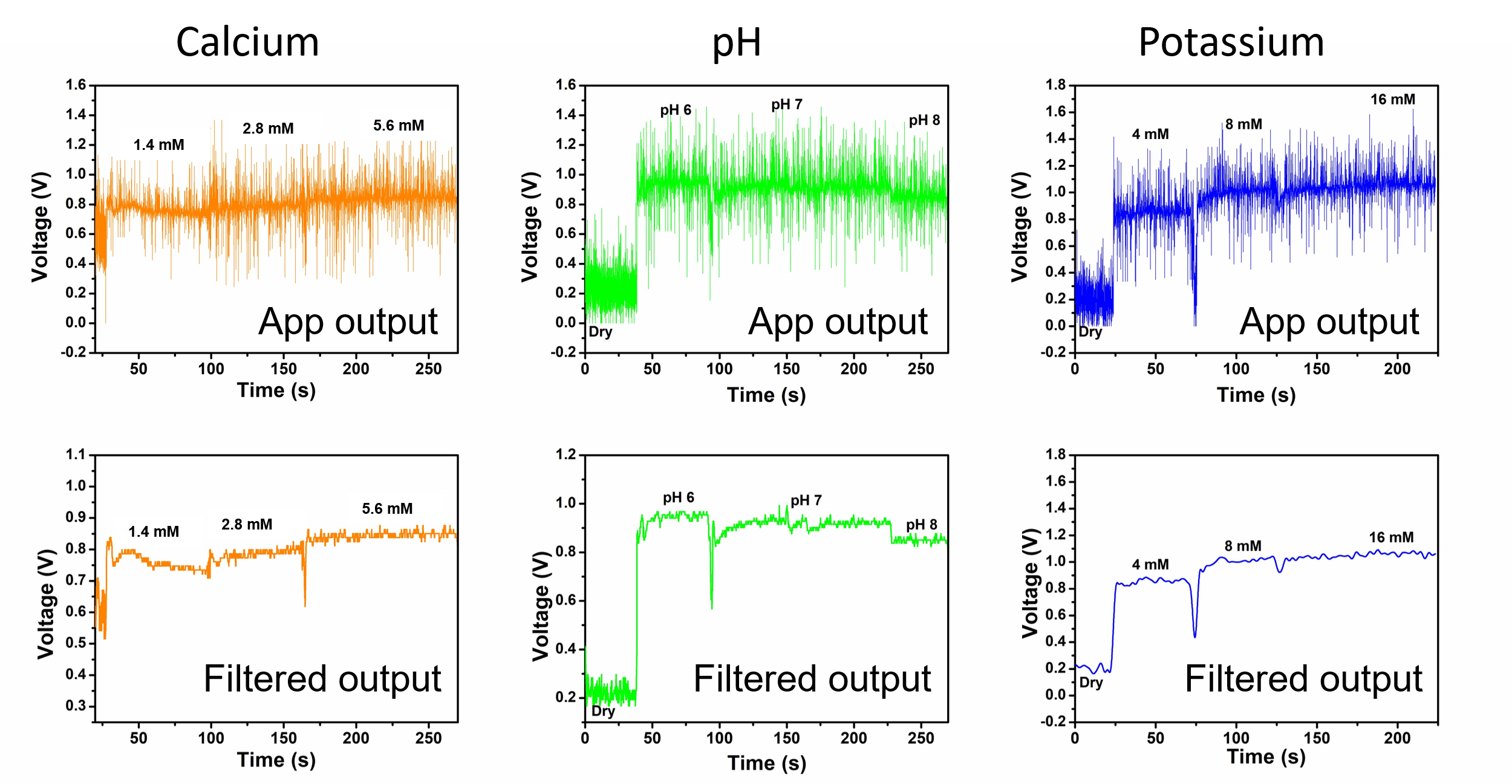


Supplementary Figure 14 | Original and filtered output of the biomarkers’ readings from the Smartphone app.

Table S1 **|** Comparison between the MMNs-EGFET sensor and other reported sensors.

| **Microneedle structure** | **Sensing methods** | **Detected analytes** | **Analytical performance** | **Reference** |
| --- | --- | --- | --- | --- |
| Solid MNs modified with Ag NWs | Extended-gate FET sensor | Na^+^, Ca^2+^, K^+^, pH | LOD Na^+^: 0.56 µM  LOD K^+^: 24.23 µM  LOD Ca^2+^: 30.96 µM  LOD pH: n/a  Sensitivity Na^+^:  3.43 mA/decade  Sensitivity K^+^:  1.15 mA/decade  Sensitivity Ca^2+^:  0.46 mA/decade  Sensitivity pH:  0.31 mA/pH | This work |
| Hollow MN | Potentiometric sensor | Na^+^, K^+^ | LOD: n/a  Sensitivity Na^+^:  56.08 mV/decade  Sensitivity K^+^:  50.03 mV/decade | [5] |
| Solid MNs | Extended-gate FET sensor | Na^+^ | LOD: 2.78 µM  Sensitivity:  5.61 mA/decade | [2] |
| Solid MNs | Potentiometric sensor | Na^+^, Ca^2+^, K^+^ | LOD: n/a  Sensitivity Ca^2+^:  21.65 mV/degree  Sensitivity K^+^:  47.1 mV/degree  Sensitivity Na^+^:  76.24 mV/degree | [6] |
| Solid MNs | Potentiometric sensor | K^+^ | LOD: 10^–4.9^  Sensitivity:  52.2 mV/decade | [3] |
| Hollow 3D-printed MN | Potentiometric sensor | pH | LOD: n/a  Sensitivity ~  67 mV/pH | [4] |
| Solid MNs | Potentiometric sensor | pH | LOD: n/a  Sensitivity ~  54 mV/decade | [7] |

**Supplementary Videos:**


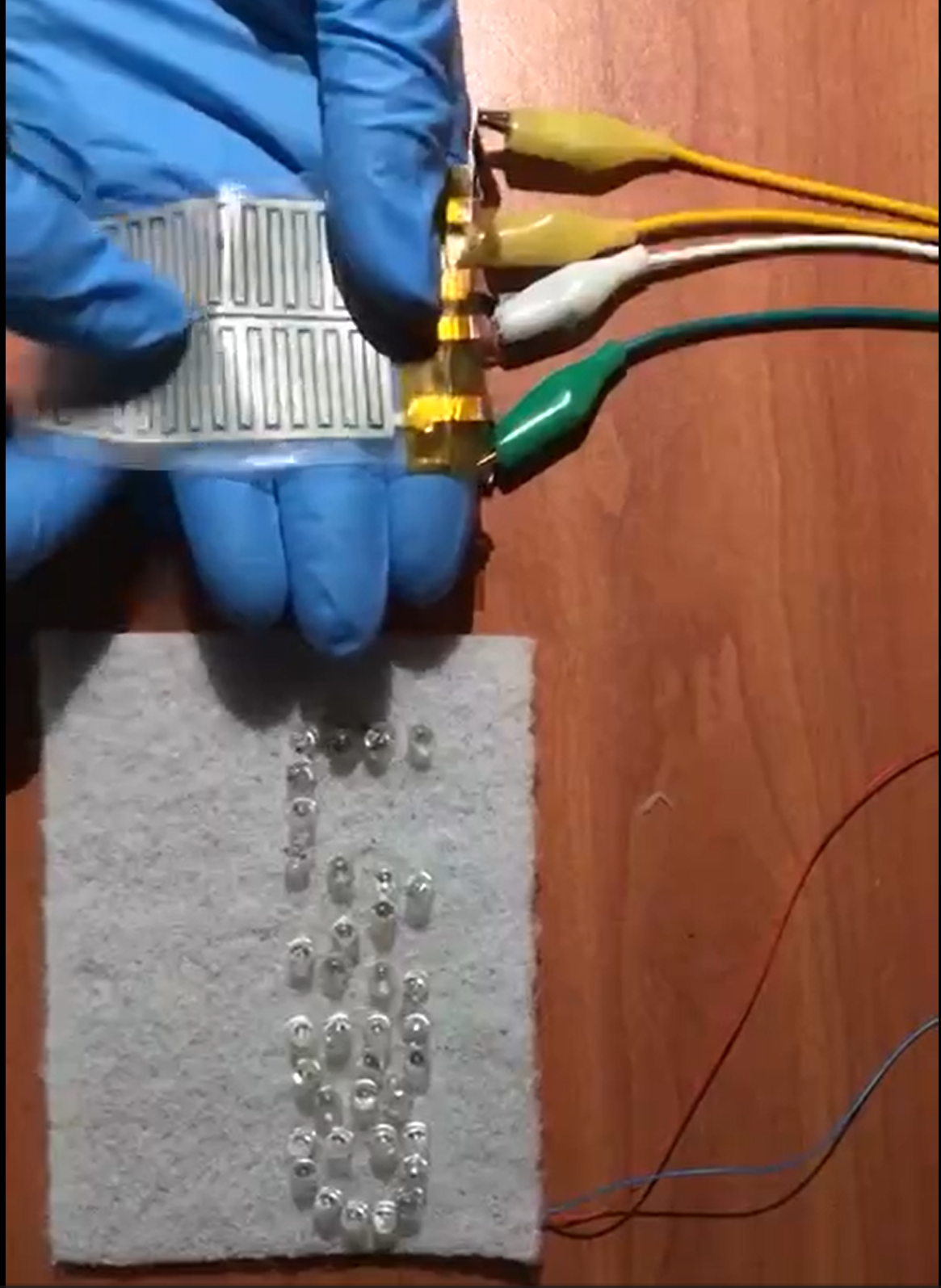


Supplementary Video 1 | Flexible TENG device and lighting LEDs


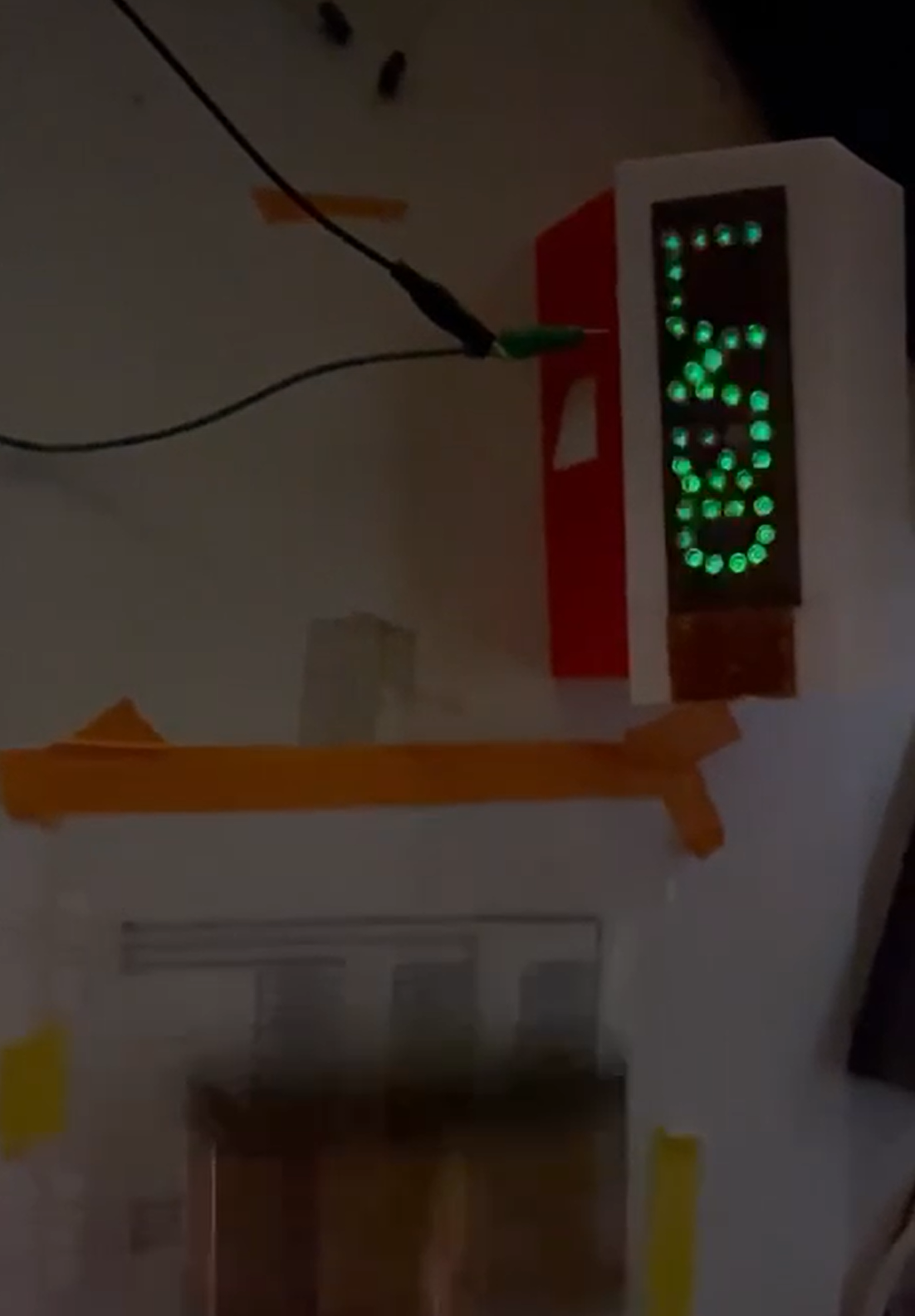


Supplementary Video 2 | Lighting LEDs using the TENG by a mechanical motor


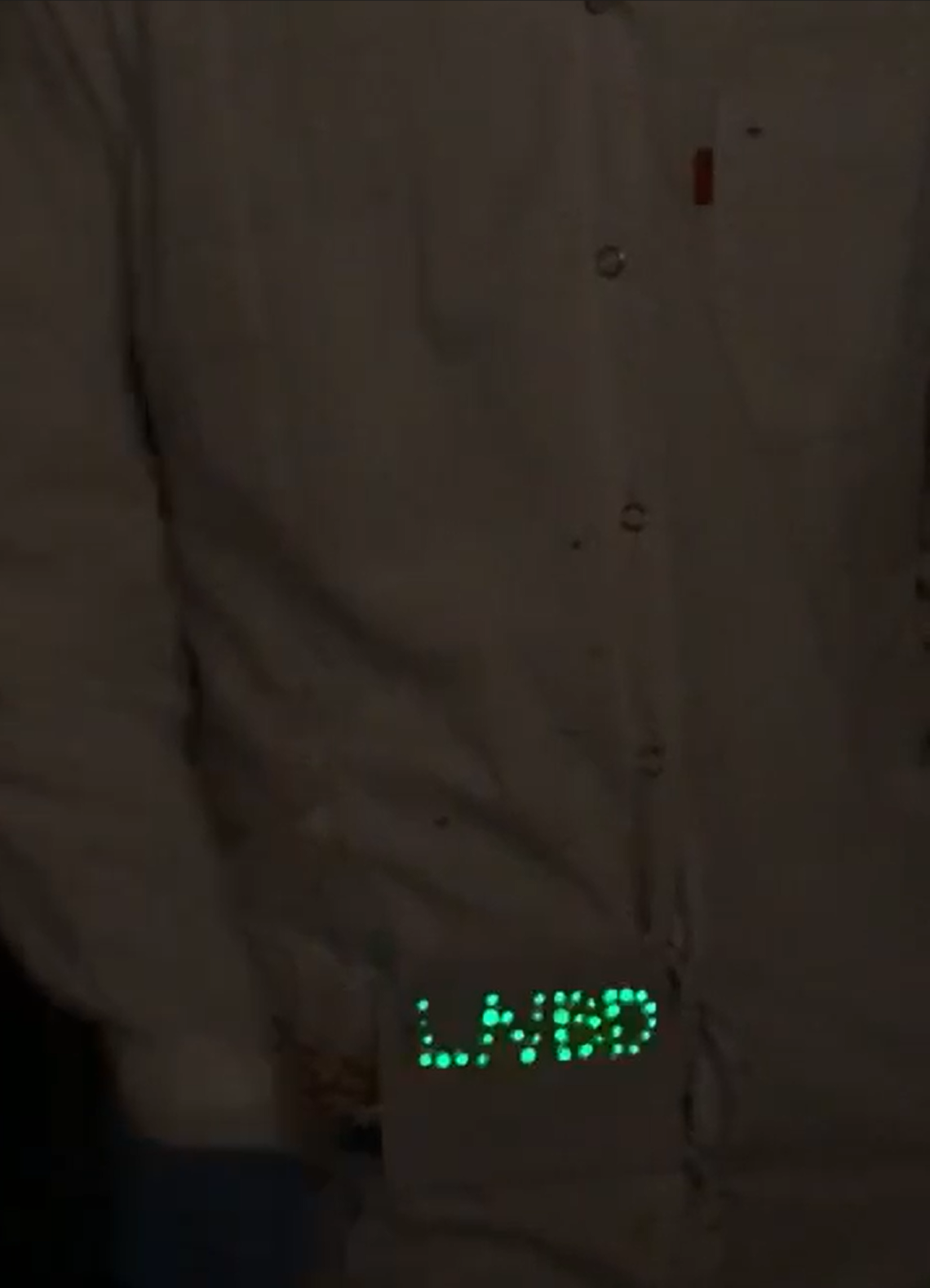


Supplementary Video 3 | Lighting LEDs using the TENG by human motion

**References**

[1] Wiley B, Sun Y, Xia Y. Polyol synthesis of silver nanostructures: control of product morphology with Fe(II) or Fe(III) species. Langmuir 2005;21:8077–80. https://doi.org/10.1021/LA050887I.

[2] Zheng Y, Omar R, Zhang R, Tang N, Khatib M, Xu Q, et al. A Wearable Microneedle-Based Extended Gate Transistor for Real-Time Detection of Sodium in Interstitial Fluids. Advanced Materials 2022;34. https://doi.org/10.1002/ADMA.202108607.

[3] Parrilla M, Cuartero M, Padrell Sánchez S, Rajabi M, Roxhed N, Niklaus F, et al. Wearable All-Solid-State Potentiometric Microneedle Patch for Intradermal Potassium Detection. Anal Chem 2019;91:1578–86. https://doi.org/10.1021/acs.analchem.8b04877.

[4] Parrilla M, Vanhooydonck A, Johns M, Watts R, De Wael K. 3D-printed microneedle-based potentiometric sensor for pH monitoring in skin interstitial fluid. Sens Actuators B Chem 2023;378:133159. https://doi.org/10.1016/J.SNB.2022.133159.

[5] Li H, Wu G, Weng Z, Sun H, Nistala R, Zhang Y. Microneedle-Based Potentiometric Sensing System for Continuous Monitoring of Multiple Electrolytes in Skin Interstitial Fluids. ACS Sens 2021;6:2181–90. https://doi.org/10.1021/acssensors.0c02330.

[6] Huang X, Zheng S, Liang B, He M, Wu F, Yang J, et al. 3D-assembled microneedle ion sensor-based wearable system for the transdermal monitoring of physiological ion fluctuations. Microsystems & Nanoengineering 2023 9:1 2023;9:1–16. https://doi.org/10.1038/s41378-023-00497-0.

[7] García-Guzmán JJ, Pérez-Ràfols C, Cuartero M, Crespo GA. Toward *In Vivo* Transdermal pH Sensing with a Validated Microneedle Membrane Electrode. ACS Sens 2021;6:1129–37. https://doi.org/10.1021/ACSSENSORS.0C02397.
